# Bis(2,4,6-trimethyl­pyridinium) tetra­bromidozincate

**DOI:** 10.1107/S1600536812040925

**Published:** 2012-10-03

**Authors:** Basem F. Ali, Salim F. Haddad, Rawhi Al-Far

**Affiliations:** a Department of Chemistry, Al al-Bayt University, Mafraq 25113, Jordan; b Department of Chemistry, The University of Jordan, Amman 11942, Jordan; c Faculty of Science and IT, Al-Balqa’a Applied University, Salt, Jordan

## Abstract

In the title compound, (C_8_H_12_N)_2_[ZnBr_4_], the coordination geometry of the anion is approximately tetra­hedral. The Zn—Br bond lengths range from 2.3901 (19) to 2.449 (2) Å and the Br—Zn—Br angles range from 107.09 (8) to 112.48 (8)°. In the crystal, each [ZnBr_4_]^2−^ anion is connected to four cations through two N—H⋯Br and two C—H⋯Br hydrogen bonds, forming two-dimensional ⋯(cation)_2_⋯anion⋯(cation_2_)⋯ sheets parallel to the *bc* plane. Within each sheet, the anions are arranged in stacks with no significant inter-anion Br⋯Br inter­actions [the shortest being > 4.3 Å], while the cations are in chains, with weak π–π stacking inter­actions [centroid–centroid distance = 3.991 Å] between cations inter­acting with the same anion.

## Related literature

For background information, see: Ali & Al-Far (2009 ▶). For bond lengths and angles in the [ZnBr_4_]^2−^ anion, see: Ali & Al-Far (2009 ▶); Peng & Li (2011 ▶). For another structure containing the 2,4,6-trimethyl­pyridinium cation, see: Abbasi *et al.* (2011 ▶).
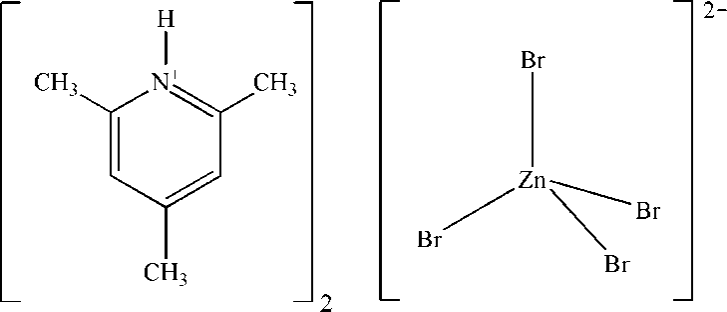


## Experimental

### 

#### Crystal data

(C_8_H_12_N)_2_[ZnBr_4_]
*M* *_r_* = 629.36
Triclinic, 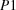

*a* = 7.3627 (8) Å
*b* = 9.0310 (8) Å
*c* = 9.1854 (9) Å
α = 101.741 (8)°
β = 110.778 (10)°
γ = 96.321 (8)°
*V* = 547.89 (9) Å^3^
*Z* = 1
Mo *K*α radiation
μ = 8.41 mm^−1^
*T* = 293 K
0.35 × 0.25 × 0.20 mm

#### Data collection

Oxford Xcalibur Eos diffractometer
Absorption correction: multi-scan (*CrysAlis PRO*; Agilent, 2011 ▶) *T*_min_ = 0.413, *T*_max_ = 1.000
3637 measured reflections
2730 independent reflections
2399 reflections with *I* > 2σ(*I*)
*R*_int_ = 0.021

#### Refinement

*R*[*F* ^2^ > 2σ(*F* ^2^)] = 0.054
*wR*(*F* ^2^) = 0.140
*S* = 1.02
2730 reflections
214 parameters
3 restraints
H-atom parameters constrained
Δρ_max_ = 0.75 e Å^−3^
Δρ_min_ = −0.77 e Å^−3^
Absolute structure: Flack (1983 ▶), 797 Friedel pairs
Flack parameter: −0.02 (2)

### 

Data collection: *CrysAlis PRO* (Agilent, 2011 ▶); cell refinement: *CrysAlis PRO*; data reduction: *CrysAlis PRO*; program(s) used to solve structure: *SHELXS97* (Sheldrick, 2008 ▶); program(s) used to refine structure: *SHELXL97* (Sheldrick, 2008 ▶); molecular graphics: *SHELXTL* (Sheldrick, 2008 ▶); software used to prepare material for publication: *SHELXTL*.

## Supplementary Material

Crystal structure: contains datablock(s) I, global. DOI: 10.1107/S1600536812040925/pv2593sup1.cif

Structure factors: contains datablock(s) I. DOI: 10.1107/S1600536812040925/pv2593Isup2.hkl

Additional supplementary materials:  crystallographic information; 3D view; checkCIF report

## Figures and Tables

**Table 1:** Hydrogen-bond geometry (Å, °)

*D*—H⋯*A*	*D*—H	H⋯*A*	*D*⋯*A*	*D*—H⋯*A*
N1—H1*A*⋯Br1^i^	0.86	2.79	3.647 (13)	175
N2—H2*A*⋯Br3	0.86	2.57	3.433 (10)	179
C2—H2*B*⋯Br3	0.93	2.79	3.685 (11)	162
C10—H10*A*⋯Br4^ii^	0.93	2.86	3.776 (13)	168

Symmetry codes: (i) 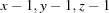; (ii) 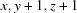.

**Fig. 1.**
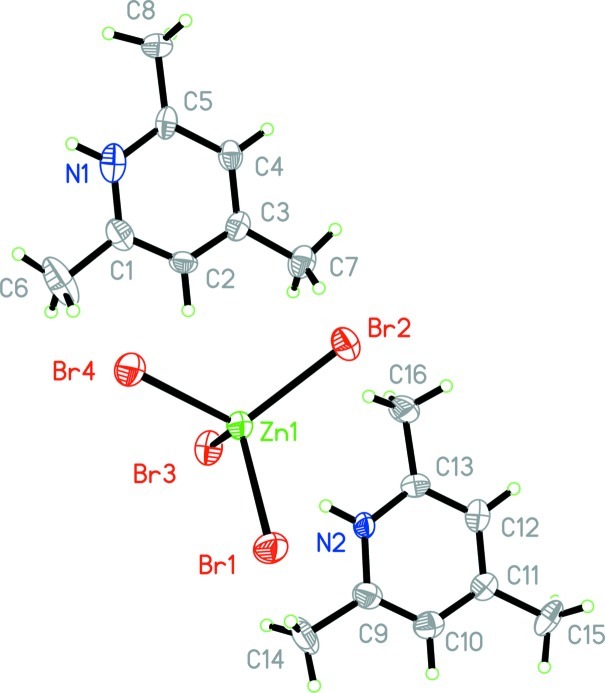
A view of the asymmetric unit of the title compound, with the atom-numbering scheme. Displacement ellipsoids are drawn at the 30% probability level.

**Fig. 2.**
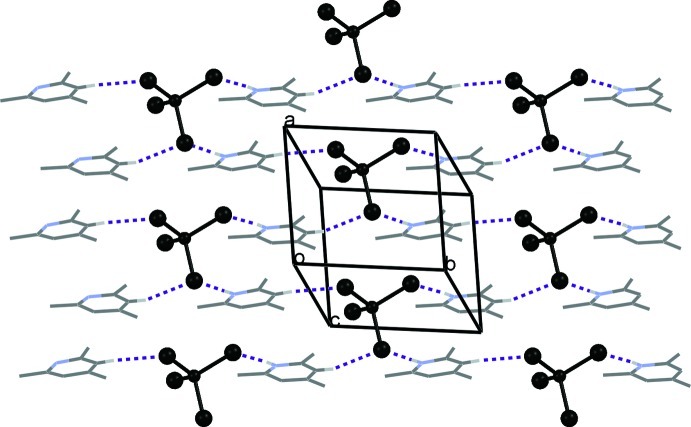
A packing diagram of the title compound showing alternating stacks of anions and cations. C/N—H···Br interactions are shown as dashed lines.

## supplementary crystallographic information

### Comment

In connection with ongoing studies of the structural aspects of halo-metal anion salts (Ali & Al-Far, 2009), we herein report the crystal structure of the title compound. The asymmetric unit contains an anion and two independent cations (Fig. 1). The geometry of ZnBr_4_^2-^ anion is approximately tetrahedral. In the anion, the bond distances and angles fall in the range of those reported previously (Peng & Li, 2011). In the cations, the bond lengths and angles are within normal ranges compared to the salt containing 2,4,6-trimethylpyridinium cation (Abbasi *et al.*, 2011). The packing of the structure can be regarded as alternating stacks of anions and chains of cations. The anion stacks are parallel to the cation chains, with no significant Br···Br interactions [shortest Br···Br interactions being greater than 4.3 Å]. The anions and cations are interacting significantly through two N—H···Br—Zn and two pyC—H···Br—Zn hydrogen bonding (Table 1). These interactions link anions and cations into two-dimensional sheets of *etc* ···(cation)_2_···anion···(cation)_2_···*etc* parallel to *bc* plane (Fig. 2).

### Experimental

To a hot solution of 2,4,6-trimethylpyridine (0.122 g, 1 mmol) and 1 ml of 60% HBr dissolved in 95% EtOH (15 ml), a hot solution of ZnCl_2_ (0.136 g, 1 mmol) dissolved in 95% EtOH (10 ml) was added. The resulting mixture was then treated with liquid Br_2_ (2 ml) and refluxed for 2 h. The resulting mixture was left undisturbed to evaporate at room temperature whereupon colorless plate crystals were formed after two days.

### Refinement

All H atoms were positioned geometrically and refined using a riding model, with N—H = 0.86 Å and C—H = 0.93 and 0.96 Å, for aryl and methyl H atoms, respectively. The *Uiso*(H) were allowed at 1.5*U*eq(C methyl) or 1.2*U*eq(N/C nonmethyl). An absolute structure was determined by using 797 Friedel pairs.

### Crystal data

**Table:**

(C_8_H_12_N)_2_[ZnBr_4_]	*Z* = 1
*M**_r_* = 629.36	*F*(000) = 304
Triclinic, *P*1	*D*_x_ = 1.908 Mg m^−^^3^
Hall symbol: P 1	Mo *K*α radiation, λ = 0.71073 Å
*a* = 7.3627 (8) Å	Cell parameters from 1674 reflections
*b* = 9.0310 (8) Å	θ = 2.9–29.1°
*c* = 9.1854 (9) Å	µ = 8.41 mm^−^^1^
α = 101.741 (8)°	*T* = 293 K
β = 110.778 (10)°	Chunk, colourless
γ = 96.321 (8)°	0.35 × 0.25 × 0.2 mm
*V* = 547.89 (9) Å^3^	

### Data collection

**Table:**

Oxford Xcalibur Eos diffractometer	2730 independent reflections
Radiation source: Enhance (Mo) X-ray Source	2399 reflections with *I* > 2σ(*I*)
Graphite monochromator	*R*_int_ = 0.021
Detector resolution: 16.0534 pixels mm^-1^	θ_max_ = 25.0°, θ_min_ = 2.9°
ω scans	*h* = −8→8
Absorption correction: multi-scan (*CrysAlis PRO*; Agilent, 2011)	*k* = −10→7
*T*_min_ = 0.413, *T*_max_ = 1.000	*l* = −10→10
3637 measured reflections	

### Refinement

**Table:**

Refinement on *F*^2^	Secondary atom site location: difference Fourier map
Least-squares matrix: full	Hydrogen site location: inferred from neighbouring sites
*R*[*F*^2^ > 2σ(*F*^2^)] = 0.054	H-atom parameters constrained
*wR*(*F*^2^) = 0.140	*w* = 1/[σ^2^(*F*_o_^2^) + (0.0933*P*)^2^] where *P* = (*F*_o_^2^ + 2*F*_c_^2^)/3
*S* = 1.02	(Δ/σ)_max_ < 0.001
2730 reflections	Δρ_max_ = 0.75 e Å^−^^3^
214 parameters	Δρ_min_ = −0.77 e Å^−^^3^
3 restraints	Absolute structure: Flack (1983), 797 Friedel pairs
Primary atom site location: structure-invariant direct methods	Flack parameter: −0.02 (2)

### Special details

**Table:**

Geometry. All e.s.d.'s (except the e.s.d. in the dihedral angle between two l.s. planes) are estimated using the full covariance matrix. The cell e.s.d.'s are taken into account individually in the estimation of e.s.d.'s in distances, angles and torsion angles; correlations between e.s.d.'s in cell parameters are only used when they are defined by crystal symmetry. An approximate (isotropic) treatment of cell e.s.d.'s is used for estimating e.s.d.'s involving l.s. planes.
Refinement. Refinement of *F*^2^ against ALL reflections. The weighted *R*-factor *wR* and goodness of fit *S* are based on *F*^2^, conventional *R*-factors *R* are based on *F*, with *F* set to zero for negative *F*^2^. The threshold expression of *F*^2^ > σ(*F*^2^) is used only for calculating *R*-factors(gt) *etc*. and is not relevant to the choice of reflections for refinement. *R*-factors based on *F*^2^ are statistically about twice as large as those based on *F*, and *R*-factors based on ALL data will be even larger.

### Fractional atomic coordinates and isotropic or equivalent isotropic displacement parameters (Å^2^)

**Table:**

	*x*	*y*	*z*	*U*_iso_*/*U*_eq_	
Zn1	0.9260 (2)	0.39402 (17)	0.49611 (17)	0.0439 (3)	
Br4	0.9532 (2)	0.27784 (15)	0.24750 (16)	0.0629 (4)	
Br3	0.59389 (18)	0.45209 (17)	0.43497 (16)	0.0576 (4)	
Br2	0.9650 (2)	0.22617 (15)	0.67076 (16)	0.0609 (4)	
Br1	1.16750 (19)	0.63427 (15)	0.62829 (16)	0.0617 (4)	
N2	0.6828 (15)	0.6293 (11)	0.8284 (12)	0.046 (2)	
H2A	0.6588	0.5839	0.7298	0.055*	
C10	0.7852 (19)	0.8528 (16)	1.0355 (17)	0.053 (3)	
H10A	0.8271	0.9595	1.0719	0.063*	
C13	0.6550 (18)	0.5429 (14)	0.9227 (15)	0.047 (3)	
C9	0.7459 (19)	0.7828 (15)	0.8773 (18)	0.052 (3)	
C12	0.7024 (18)	0.6132 (16)	1.0853 (15)	0.053 (3)	
H12A	0.6917	0.5538	1.1546	0.064*	
C11	0.7654 (18)	0.7721 (14)	1.1426 (16)	0.045 (3)	
N1	0.2717 (18)	−0.1748 (16)	0.0480 (15)	0.065 (3)	
H1A	0.2518	−0.2148	−0.0511	0.078*	
C4	0.2765 (16)	−0.1995 (15)	0.3001 (15)	0.046 (3)	
H4A	0.2619	−0.2619	0.3658	0.056*	
C2	0.3449 (17)	0.0429 (13)	0.2619 (15)	0.045 (3)	
H2B	0.3782	0.1497	0.3019	0.054*	
C5	0.2520 (17)	−0.2679 (15)	0.1393 (14)	0.045 (3)	
C3	0.3220 (18)	−0.0405 (15)	0.3603 (15)	0.047 (3)	
C1	0.323 (2)	−0.0168 (17)	0.1074 (18)	0.058 (3)	
C15	0.804 (2)	0.848 (2)	1.3147 (17)	0.067 (4)	
H15A	0.9121	0.8130	1.3848	0.100*	
H15B	0.6876	0.8221	1.3356	0.100*	
H15C	0.8381	0.9580	1.3338	0.100*	
C7	0.350 (2)	0.038 (2)	0.5270 (19)	0.062 (4)	
H7A	0.4814	0.0996	0.5823	0.093*	
H7B	0.3321	−0.0378	0.5830	0.093*	
H7C	0.2547	0.1030	0.5239	0.093*	
C14	0.774 (3)	0.864 (2)	0.758 (2)	0.083 (5)	
H14A	0.8654	0.8205	0.7171	0.125*	
H14B	0.8253	0.9715	0.8091	0.125*	
H14C	0.6487	0.8504	0.6700	0.125*	
C8	0.202 (2)	−0.4391 (14)	0.0714 (18)	0.057 (3)	
H8A	0.1790	−0.4631	−0.0411	0.086*	
H8B	0.0841	−0.4806	0.0842	0.086*	
H8C	0.3094	−0.4835	0.1277	0.086*	
C16	0.585 (3)	0.3720 (16)	0.851 (2)	0.064 (4)	
H16A	0.6220	0.3414	0.7604	0.097*	
H16B	0.4432	0.3467	0.8150	0.097*	
H16C	0.6444	0.3188	0.9303	0.097*	
C6	0.353 (4)	0.090 (3)	0.010 (3)	0.120 (9)	
H6A	0.2549	0.1531	−0.0043	0.179*	
H6B	0.3405	0.0304	−0.0941	0.179*	
H6C	0.4828	0.1540	0.0641	0.179*	

### Atomic displacement parameters (Å^2^)

**Table:**

	*U*^11^	*U*^22^	*U*^33^	*U*^12^	*U*^13^	*U*^23^
Zn1	0.0515 (8)	0.0429 (7)	0.0372 (7)	0.0055 (6)	0.0178 (6)	0.0109 (5)
Br4	0.0890 (11)	0.0564 (9)	0.0506 (8)	0.0112 (7)	0.0382 (8)	0.0105 (6)
Br3	0.0525 (8)	0.0733 (9)	0.0461 (7)	0.0155 (7)	0.0172 (6)	0.0155 (6)
Br2	0.0802 (10)	0.0601 (8)	0.0521 (8)	0.0160 (7)	0.0292 (7)	0.0279 (7)
Br1	0.0650 (9)	0.0521 (8)	0.0573 (9)	−0.0076 (6)	0.0207 (7)	0.0073 (6)
N2	0.055 (6)	0.047 (6)	0.029 (5)	−0.006 (5)	0.012 (4)	0.012 (4)
C10	0.042 (6)	0.048 (7)	0.061 (9)	0.009 (5)	0.013 (6)	0.009 (6)
C13	0.047 (7)	0.037 (6)	0.053 (7)	0.002 (5)	0.019 (6)	0.007 (6)
C9	0.051 (7)	0.047 (8)	0.059 (8)	0.007 (6)	0.020 (6)	0.018 (6)
C12	0.049 (7)	0.070 (9)	0.041 (7)	0.016 (7)	0.017 (6)	0.015 (6)
C11	0.036 (6)	0.051 (8)	0.047 (7)	0.009 (5)	0.016 (5)	0.008 (6)
N1	0.059 (7)	0.094 (10)	0.044 (6)	0.012 (6)	0.020 (5)	0.021 (6)
C4	0.041 (6)	0.062 (8)	0.040 (6)	0.017 (6)	0.016 (5)	0.018 (6)
C2	0.047 (7)	0.029 (6)	0.051 (8)	0.003 (5)	0.011 (6)	0.010 (5)
C5	0.035 (6)	0.069 (8)	0.030 (6)	0.011 (5)	0.010 (5)	0.013 (6)
C3	0.045 (7)	0.058 (8)	0.037 (6)	0.015 (6)	0.014 (5)	0.007 (6)
C1	0.051 (8)	0.064 (9)	0.058 (9)	0.007 (6)	0.014 (6)	0.031 (7)
C15	0.064 (9)	0.082 (11)	0.037 (8)	0.007 (8)	0.018 (7)	−0.014 (7)
C7	0.066 (10)	0.074 (10)	0.048 (8)	0.020 (8)	0.027 (7)	0.009 (7)
C14	0.109 (14)	0.086 (12)	0.073 (12)	0.029 (10)	0.035 (10)	0.057 (10)
C8	0.070 (9)	0.037 (7)	0.061 (9)	0.006 (6)	0.024 (7)	0.009 (6)
C16	0.087 (11)	0.049 (8)	0.065 (10)	0.000 (7)	0.039 (8)	0.021 (7)
C6	0.120 (17)	0.15 (2)	0.119 (19)	0.027 (15)	0.047 (15)	0.101 (17)

### Geometric parameters (Å, º)

**Table:**

Zn1—Br2	2.3901 (19)	C2—C1	1.356 (19)
Zn1—Br4	2.398 (2)	C2—H2B	0.9300
Zn1—Br1	2.4270 (19)	C5—C8	1.494 (18)
Zn1—Br3	2.449 (2)	C3—C7	1.480 (19)
N2—C13	1.329 (16)	C1—C6	1.49 (2)
N2—C9	1.340 (16)	C15—H15A	0.9600
N2—H2A	0.8600	C15—H15B	0.9600
C10—C9	1.37 (2)	C15—H15C	0.9600
C10—C11	1.38 (2)	C7—H7A	0.9600
C10—H10A	0.9300	C7—H7B	0.9600
C13—C12	1.397 (18)	C7—H7C	0.9600
C13—C16	1.501 (18)	C14—H14A	0.9600
C9—C14	1.50 (2)	C14—H14B	0.9600
C12—C11	1.387 (18)	C14—H14C	0.9600
C12—H12A	0.9300	C8—H8A	0.9600
C11—C15	1.498 (18)	C8—H8B	0.9600
N1—C5	1.333 (18)	C8—H8C	0.9600
N1—C1	1.377 (19)	C16—H16A	0.9600
N1—H1A	0.8600	C16—H16B	0.9600
C4—C3	1.385 (17)	C16—H16C	0.9600
C4—C5	1.418 (17)	C6—H6A	0.9600
C4—H4A	0.9300	C6—H6B	0.9600
C2—C3	1.331 (18)	C6—H6C	0.9600
			
Br2—Zn1—Br4	112.48 (8)	C2—C1—N1	117.5 (12)
Br2—Zn1—Br1	110.92 (8)	C2—C1—C6	119.3 (16)
Br4—Zn1—Br1	109.19 (7)	N1—C1—C6	123.1 (16)
Br2—Zn1—Br3	107.09 (8)	C11—C15—H15A	109.5
Br4—Zn1—Br3	108.55 (8)	C11—C15—H15B	109.5
Br1—Zn1—Br3	108.49 (8)	H15A—C15—H15B	109.5
C13—N2—C9	124.2 (11)	C11—C15—H15C	109.5
C13—N2—H2A	117.9	H15A—C15—H15C	109.5
C9—N2—H2A	117.9	H15B—C15—H15C	109.5
C9—C10—C11	122.8 (12)	C3—C7—H7A	109.5
C9—C10—H10A	118.6	C3—C7—H7B	109.5
C11—C10—H10A	118.6	H7A—C7—H7B	109.5
N2—C13—C12	118.9 (11)	C3—C7—H7C	109.5
N2—C13—C16	118.0 (11)	H7A—C7—H7C	109.5
C12—C13—C16	123.1 (12)	H7B—C7—H7C	109.5
N2—C9—C10	116.9 (12)	C9—C14—H14A	109.5
N2—C9—C14	117.8 (13)	C9—C14—H14B	109.5
C10—C9—C14	125.2 (14)	H14A—C14—H14B	109.5
C11—C12—C13	119.6 (12)	C9—C14—H14C	109.5
C11—C12—H12A	120.2	H14A—C14—H14C	109.5
C13—C12—H12A	120.2	H14B—C14—H14C	109.5
C10—C11—C12	117.4 (12)	C5—C8—H8A	109.5
C10—C11—C15	123.1 (12)	C5—C8—H8B	109.5
C12—C11—C15	119.5 (13)	H8A—C8—H8B	109.5
C5—N1—C1	122.0 (12)	C5—C8—H8C	109.5
C5—N1—H1A	119.0	H8A—C8—H8C	109.5
C1—N1—H1A	119.0	H8B—C8—H8C	109.5
C3—C4—C5	120.7 (12)	C13—C16—H16A	109.5
C3—C4—H4A	119.7	C13—C16—H16B	109.5
C5—C4—H4A	119.7	H16A—C16—H16B	109.5
C3—C2—C1	124.6 (12)	C13—C16—H16C	109.5
C3—C2—H2B	117.7	H16A—C16—H16C	109.5
C1—C2—H2B	117.7	H16B—C16—H16C	109.5
N1—C5—C4	118.0 (12)	C1—C6—H6A	109.5
N1—C5—C8	120.4 (12)	C1—C6—H6B	109.5
C4—C5—C8	121.6 (12)	H6A—C6—H6B	109.5
C2—C3—C4	117.0 (11)	C1—C6—H6C	109.5
C2—C3—C7	119.7 (12)	H6A—C6—H6C	109.5
C4—C3—C7	123.3 (13)	H6B—C6—H6C	109.5
			
C9—N2—C13—C12	−3.1 (18)	C1—N1—C5—C4	2.9 (19)
C9—N2—C13—C16	−179.7 (13)	C1—N1—C5—C8	−178.3 (13)
C13—N2—C9—C10	0.6 (19)	C3—C4—C5—N1	−0.8 (17)
C13—N2—C9—C14	178.8 (13)	C3—C4—C5—C8	−179.6 (12)
C11—C10—C9—N2	1 (2)	C1—C2—C3—C4	1.3 (19)
C11—C10—C9—C14	−177.0 (14)	C1—C2—C3—C7	179.9 (13)
N2—C13—C12—C11	3.9 (18)	C5—C4—C3—C2	−1.3 (17)
C16—C13—C12—C11	−179.7 (13)	C5—C4—C3—C7	−179.8 (12)
C9—C10—C11—C12	−0.1 (19)	C3—C2—C1—N1	1 (2)
C9—C10—C11—C15	−178.8 (13)	C3—C2—C1—C6	−180.0 (15)
C13—C12—C11—C10	−2.4 (17)	C5—N1—C1—C2	−3 (2)
C13—C12—C11—C15	176.3 (12)	C5—N1—C1—C6	177.8 (15)

### Hydrogen-bond geometry (Å, º)

**Table:**

*D*—H···*A*	*D*—H	H···*A*	*D*···*A*	*D*—H···*A*
N1—H1*A*···Br1^i^	0.86	2.79	3.647 (13)	175
N2—H2*A*···Br3	0.86	2.57	3.433 (10)	179
C2—H2*B*···Br3	0.93	2.79	3.685 (11)	162
C10—H10*A*···Br4^ii^	0.93	2.86	3.776 (13)	168

Symmetry codes: (i) *x*−1, *y*−1, *z*−1; (ii) *x*, *y*+1, *z*+1.
